# pH-responsive cinnamaldehyde-TiO_2_ nanotube coating: fabrication and functions in a simulated diabetes condition

**DOI:** 10.1007/s10856-022-06683-2

**Published:** 2022-09-05

**Authors:** Yichen Lee, Jingyan Huang, Zhaoxia Bing, Kaiting Yuan, Jinghong Yang, Min Cai, Shiqi Zhou, Bo Yang, Wei Teng, Weichang Li, Yan Wang

**Affiliations:** 1 Hospital of Stomatology, Sun Yat-sen University, Guangzhou, 510055 PR China; 2 Guangdong Provincial Key Laboratory of Stomatology, Guangzhou, 510055 PR China

## Abstract

Current evidence has suggested that diabetes increases the risk of implanting failure, and therefore, appropriate surface modification of dental implants in patients with diabetes is crucial. TiO_2_ nanotube (TNT) has an osteogenic nanotopography, and its osteogenic properties can be further improved by loading appropriate drugs. Cinnamaldehyde (CIN) has been proven to have osteogenic, anti-inflammatory, and anti-bacterial effects. We fabricated a pH-responsive cinnamaldehyde-TiO_2_ nanotube coating (TNT-CIN) and hypothesized that this coating will exert osteogenic, anti-inflammatory, and anti-bacterial functions in a simulated diabetes condition. TNT-CIN was constructed by anodic oxidation, hydroxylation, silylation, and Schiff base reaction to bind CIN, and its surface characteristics were determined. Conditions of diabetes and diabetes with a concurrent infection were simulated using 22-mM glucose without and with 1-μg/mL lipopolysaccharide, respectively. The viability and osteogenic differentiation of bone marrow mesenchymal stem cells, polarization and secretion of macrophages, and resistance to *Porphyromonas gingivalis* and *Streptococcus mutans* were evaluated. CIN was bound to the TNT surface successfully and released better in low pH condition. TNT-CIN showed better osteogenic and anti-inflammatory effects and superior bacterial resistance than TNT in a simulated diabetes condition. These findings indicated that TNT-CIN is a promising, multifunctional surface coating for patients with diabetes needing dental implants.

**Figure:**
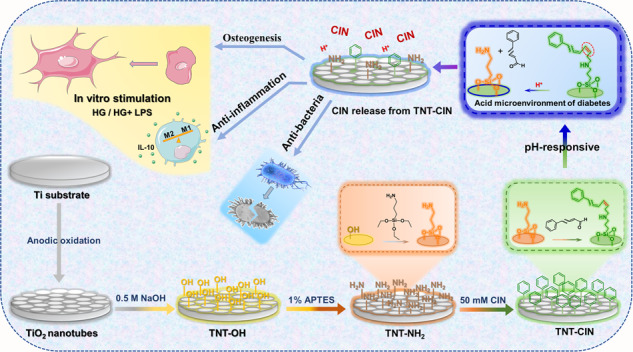
Graphical abstract

Graphical abstract

## Introduction

The global prevalence of diabetes has been increasing in the past decades and is estimated to rise to 10.2% (578 million) by 2030 and 10.9% (700 million) by 2045 [1]. Because of the advanced glycation end products (AGES) accumulated in diabetes, the bone marrow microenvironment gets disrupted [2, 3] and the proinflammatory signaling cascades are activated, thus inhibiting osteogenesis and promoting osteoclast activity [3]. The concentration of AGES in peri-implant sulcular fluid is reportedly significantly correlated with pocket depth and jaw bone loss around implants [4]. Conversely, diabetes increases the susceptibility to developing opportunistic infections. Current evidence suggests that compared with healthy population, patients with hyperglycemia are at an approximately 50% higher risk for developing peri-implantitis [5]. Lipopolysaccharide (LPS) is a toxic component produced by bacteria and has a strong destructive effect on periodontal tissues [6]. Furthermore, LPS aggravates the injury caused by hyperglycemia [7]. Briefly, diabetes caused chronic persistent inflammation easily and thus has been regarded as a relative contraindication for dental implants.

Many researches have focused on surface modification of implants to improve the success rate of implants in patients with diabetes. One of the modification strategies is to change implant surface topography. Nano-scale topography reportedly enhances osteogenic differentiation through mechanotransduction pathways involving integrin signaling, actin cytoskeleton reorganization, and nuclear mechanotransduction [8, 9]. TiO_2_ nanotube arrays (TNT), which have a regular and highly ordered nanotopography, have been proven to have extraordinary osteogenesis ability [10] even in a diabetes rat model [11]. However, TNT itself does not have any biofunction, and therefore, improving the anti-inflammatory and antibacterial effects of TNTs is important.

To achieve the abovementioned combined functions (osteogenic, anti-inflammatory, and antibacterial functions), loading a suitable drug on TNT may be a potential solution. TNT can be used as favorable drug carrier because of its high surface-to-volume ratio [9, 12]. Previously, loading of peptides [13], proteins [14], and nanoparticles [15] on TNT has been attempted; However, most of them are expensive, hard to preserve [16], and have a risk of adverse effects because of their narrow range of safe and effective concentration [17]. In the present, plant-derived extracts have gradually attracted the attention of researchers for their properties of better safety, ease of procurement, and fewer adverse effects [18]. Cinnamon oil, which is derived from the bark of trees belonging to the genus Cinnamomum, has been widely used as a flavoring agent and additive for centuries in the food industry. It has been reported to have antidiabetic, antibacterial, antifungal, anti-cancer, and anti-inflammatory effects [18–21]. Cinnamaldehyde (CIN) is one of the main resinous effective ingredients of cinnamon. In recent years, CIN has been reported to have diverse anti-diabetes [22, 23], osteogenic [24–26], anti-inflammatory [27, 28], and anti-bacterial effect [19, 29] and has been applied in oral care products because of its pleasant smell and potent anti-bacterial effects.

To improve the success rate of dental implant treatment in patients with diabetes, in this study, a biofunctional surface coating modification of Ti was explored by coating CIN onto the TNT surface via Schiff base bonds, which can be speedily hydrolyzed in the acidic microenvironment caused by diabetes. Then, the surface characteristics and release kinetics were investigated, and the osteogenic, anti-inflammatory, and antibacterial effects of the coating in a simulated diabetes condition were evaluated.

## Materials and methods

### Fabrication and surface characterization of TNT and TNT-CIN

#### Specimen fabrication

**Scheme 1:**
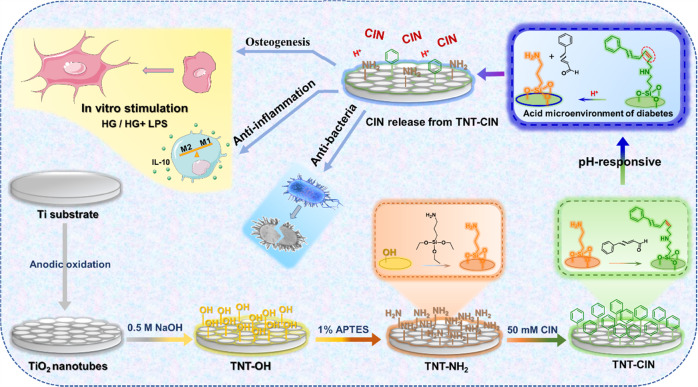
The schematic showed the construction of pH-responsive cinnamaldehyde-TiO_2_ nanotube coating (TNT-CIN), and illustrated its anti-inflammatory, osteogenic, and antibacterial effects in a simulated diabetes condition

Ti discs (dimensions: 10.0 × 10.0 × 0.3 mm^3^ or 20.0 × 20.0 × 0.3 mm^3^ for 48- or 12-well plates, respectively) obtained from Baoji Titanium Industry, China, were used in this experiment. The discs grouped in TNT were successively polished with #400, #1000, and #1500 SiC sandpapers gradually and sequentially and then fabricated by the anodic oxidation method by soaking them in an anodizing solution containing 0.15-M NH_4_F and 0.5-M (NH_4_)_2_SO_4_ at 20 V for 30 min [10]. As for the TNT-CIN group, half of the abovementioned anodic oxidized discs were randomly chosen to further covalently bind with CIN. TNT discs were placed in a 50-mM NaOH solution for 15 min, soaked in a 1% 3-aminopropyltriethoxysilane (APTES) anhydrous ethanol solution while stirring for 2 h, and then immersed in 0.5-M cinnamaldehyde anhydrous ethanol solution at 60 °C for 24 h (Scheme 1).

#### Characterization of TNT and TNT-CIN

The surface topography of the Ti specimen was examined under a scanning electron microscope (Sigma 300, Zeiss, Germany). The surface chemical functional groups were analyzed by Fourier transform infrared spectroscopy (Nicolet6700-Contiuμm, Thermo Scientific, America). Hydrophilicity of the titanium surface was evaluated using a contact angle analyzer (OCA15, Dataphysics, Germany). The surface roughness of samples was measured using a laser scanning confocal microscope profilometer (LSM700, Zeiss, Germany). Since the pH during inflammation could be lowered to 5.4 relative to a pH of 7.4 in physiological condition [30], the OD value at λ = 280 nm was detected using a spectrophotometer (NanoDrop™ one, Thermo Scientific, America) at pH values of 5.4 and 7.4 at specific time points. Cumulative release of CIN was calculated after converting the OD value into concentration according to the standard curve.

### Cell culture and simulation of diabetic condition

Bone marrow mesenchymal stem cells (BMSCs) were harvested from 3-week-old male C57BL/6 mice. Briefly, the hind limbs were cut into 2-mm fragments after the bone marrow was flushed out. The fragments were put into 1-mg/mL collagenase II (Gibco) complete medium (a-MEM containing 10% fetal bovine serum along with 1% penicillin and streptomycin) for 2 h in a humidified atmosphere of 5% CO_2_ at 37 °C, and after 2 h, they were transferred to a fresh complete medium. The medium was changed every 2–3 days. The cells were digested using 0.25% trypsin-EDTA solution (Gibco) when 85% confluence was reached; then, the cells were used in the following in vitro experiments at passage 2–4. Mineralizing medium (complete medium supplemented with 0.1-μM dexamethasone, 50-μg/mL ascorbic acid, and 10-mM β-glycerophosphate) was used for osteogenic induction for 7, 14, or 21 days.

RAW 264.7 cells (Mϕ, a murine-derived macrophage cell line kindly provided by the Institute of Biochemistry and Cell Biology, Chinese Academy of Sciences, Shanghai, China) were cultured in low-glucose DMEM (Life Technologies) according to a standard protocol [31].

Next, 22-mM glucose and 22-mM glucose + with 1-μg/mL PG-LPS (InvivoGEN, USA) were added to complete medium and mineralizing medium to simulate diabetes and diabetes complicated with infection in vitro, respectively; these groups were named the high-glucose (HG) and HG + LPS group, respectively. Treatment with 5.5-mM glucose without LPS represented the control group. These groups were used in the following experiments aimed at evaluating the osteogenic and anti-inflammatory effects of CIN.

Under anaerobic condition (80% N_2_, 10% CO_2_, 10% H_2_) at 37 °C, *Streptococcus mutans* (*S. mutans* ATCC 25175, from Guangdong Microbial Culture Collection Center, China) were cultured in brain heart infusion (BHI) broth and passaged every day. Meanwhile, Gram-negative *Porphyromonas gingivalis* strain (*P. gingivalis* ATCC33177, from Guangdong Microbial Culture Collection Center, China) was cultured in BHI broth supplemented with L-cysteine (5 μg/mL), vitamin K1 (1 μg/mL), yeast, and hemin chloride, and need a passage culture every two days. Cells in logarithmic growth phase obtained from passages 2–3 from both cultures were used for the following in vitro experiments.

### Cell proliferation and adhesion

Cell proliferation was determined using the Cell Counting Kit-8 (CCK-8) assay (Dojindo, Kumamoto, Japan). BMSCs were seeded on different Ti surfaces in different culture medium. The viability at 1, 4, and 7 days was determined using the CCK-8 assay according to the manufacturer’s instructions. The adhesion to surfaces was evaluated by observing the spread of the cytoskeleton. After one-day culture, cellular F-actin was stained with fluorescein isothiocyanate (FITC)-labeled phalloidin (Sigma) and the nucleus was stained with DAPI; subsequently, the cells were observed by confocal laser scanning microscopy. At last, cell area was measured by image J.

### Evaluation of osteogenic ability on TNT and TNT-CIN surfaces in vitro

To evaluate the osteogenic ability of BMSCs on TNT and TNT-CIN surfaces in HG and HG + LPS groups, the expression of osteogenic proteins, including alkaline phosphatase (ALP), runt-related transcription factor-2 (Runx-2), and osteopontin (OPN), was detected by immunofluorescence. The cells were subjected to the culture of 7 and 14 days and then fixed with 4% paraformaldehyde, treated with 0.1% Triton X-100 for 20 min, and sealed with 5% bovine serum albumin for 1 h. Then, the cells cultured for 7 days were incubated with mouse anti-mouse ALP (1:250, Santa Cruz, USA) or rabbit anti-mouse Runx-2 (1:250, Affinity, USA) at 4 °C for 16 h, whereas those cultured for 14 days were incubated with mouse anti-mouse ALP (1:250, Santa Cruz, USA) or rabbit anti-mouse OPN (1:250, Affinity, USA) at 4 °C for 16 h. Next, the cells were incubated with rabbit anti-mouse IgG-Alexa Fluor 594 (1:250, Bioss, China), donkey anti-rabbit IgG-AlexaFluor 488 (1:250, Absin, China), or donkey anti-rabbit IgG-Alexa Fluor 555 (1:250, Absin, China) for 1 h. Finally, cell nuclei were stained with DAPI in a dark room for 5 min. Fluorescence intensity was observed by confocal scanning laser microscopy (FV3000, Olympus, Japan) and semi-quantitatively analyzed by Image J (V2.1.4.7, National Institutes of Health, USA).

In addition, Western blot analysis of ALP, osteocalcin (OCN) were carried out after osteogenic induction for 14 days. BMSCs on the titanium surface were washed with PBS at 4 °C, each group was totally added 100 μL RIPA lysis buffer (CWBIO, China) containing 50 mM Tris, 150 mM NaCl, 1% Triton X-100, 1% sodium deoxycholate and 0.1% SDS, reacting on ice for 30 min. The lysate was centrifugated and the supernatant was collected. Protein extracts were separated by SDS-PAGE, transferred to PVDF membrane, and then probed with 1:1000 rabbit anti-ALP (Affinity, USA) and 1:1000 rabbit anti-OCN (Affinity, USA). After overnight incubation at 4 °C with shaking, 1:10,000 goat anti-rabbit HRP-conjugated IgG (Emarbio Science &Technology Company, China) was added for another 1 h incubation. Finally, proteins were displayed with ECL Plus Detection Kits (Boster, China).

The relative mRNA expression of Runx-2, OCN, ALP, and collagen-1(Col-1) during osteogenesis was also detected by PCR. Briefly, after osteogenic induction of 14 days, RNA from each group of cells was extracted using the RNA-Quick Purification Kit (Yishan, Shanghai, China) following the manufacturer’s protocol. cDNA was obtained by reverse transcription using the protocol of TB Green^TM^ Premix Ex Taq^TM^2 (Takara, Japan). The sequences of the primers used for PCR are provided in Table S1. Data were collected and analyzed using the ABI Quantstudio 5 instrument (Applied Biosystems Life Technologies, Carlsbad, USA).

Osteogenic ability of BMSCs was also evaluated by alizarin red staining. After incubation for 21 days, cells were fixed with 4% paraformaldehyde, stained with the alizarin red solution (Cyagen, Santa Clara, CA, USA). Images of the stained cells were acquired using a digital microscope (M205A, Leica, Germany). Then, alizarin red- stained nodes on the specimens were dissolved with 10% cetylpyridinium chloride (Sigma), and absorbance was measured at 562 nm for semiquantitative analysis.

### Evaluation of anti-inflammatory ability on TNT and TNT-CIN surfaces in vitro

The expressions of the M1 marker iNOS and the M2 marker CD163 were evaluated by immunofluorescent staining assays to evaluate the polarization of macrophages. iNOS and CD163 were labeled with rabbit anti-mouse iNOS (1:250, Abcam, USA), mouse anti-mouse CD163 (1:250, Bioss, China), and donkey anti-rabbit Alexa Fluor 555 (1:250, Bioss, China) and simultaneously stained with mouse anti-mouse CD163 (1:250, Bioss, China). The analysis of immunofluorescence intensity was performed as previously described.

The relative mRNA expression of anti-inflammatory cytokines was also detected by PCR with the abovementioned method. The sequences of the primers of IL-18, CD206, IL-10 and iNOS are provided in Table S1.

TNF-α, NF-kB, and IL-10, which are inflammatory-associated cytokines, were detected by enzyme-linked immunosorbent assay kits (Cusabio, Wuhan, China). After culture for 3 days as previously described, the supernatants of macrophages were collected after centrifugation at 1000 *g* for 10 min. Concentrations of TNF-α, NF-kB, IL-10 in the supernatant were quantified using an enzyme-labeled instrument (Epoch; BioTek Instruments, Inc., Winooski, VT, USA) according to the manufacturer’s instructions.

### Evaluation of anti-bacteria ability on TNT and TNT-CIN surfaces in vitro

The morphology of *S. mutans* and *P. gingivalis* was observed using a scanning electron microscope. After culture for 1 day, the Ti discs were washed with PBS and fixed for 6 h by 2.5 % glutaraldehyde solution. Then the samples were dehydrated using graded ethanol, dried and at last observed by scanning electron microscope.

Bactericidal capacity was examined using LIVE/DEAD® BacLight Bacterial Viability Kits (Thermo Fisher Scientific, USA). Briefly, samples were gently washed using deionized water after incubating for 1 day on TNT and TNT-CIN surfaces. Then, SYTO 9 dye and propidium iodide were mixed in a 1:1 ratio and diluted 3000× with deionized water as the working fluid. Samples were incubated with the working fluid for 15 min at room temperature. Alive bacteria were labeled with a green fluorescence stain (propodium iodide), whereas dead bacteria were labeled with a red fluorescence dye (Syto 9) under confocal laser scanning microscopy (FV3000, Olympus, Japan). Semi-quantitative analysis was carried out as previously described.

The number of alive bacteria on different surfaces was also measured using the plate counting method. Samples were washed by PBS. Then, the Ti discs were processed with ultrasound in 1 mL of PBS to gather the bacteria on the Ti discs. After 1 mL of bacterial suspension was diluted 1000×, 20 μL of the diluent was evenly spread on the solid medium. Finally, bacterial colonies were counted after culture for 24 h

### Statistical analysis

Statistical analysis was conducted using GraphPad Prism 8.0.1 (Graphpad Software, La Jolla, CA, USA). Continuous variables were expressed as mean ± standard deviation. After the normal distribution test, between-group differences were assessed using one-way analysis of variance followed by the least significant difference test. *P* values < 0.05 were considered indicative of statistical significance.

## Results

### pH-responsive TNT-CIN was successfully fabricated and had similar characteristics as TNT

**Fig. 1:**
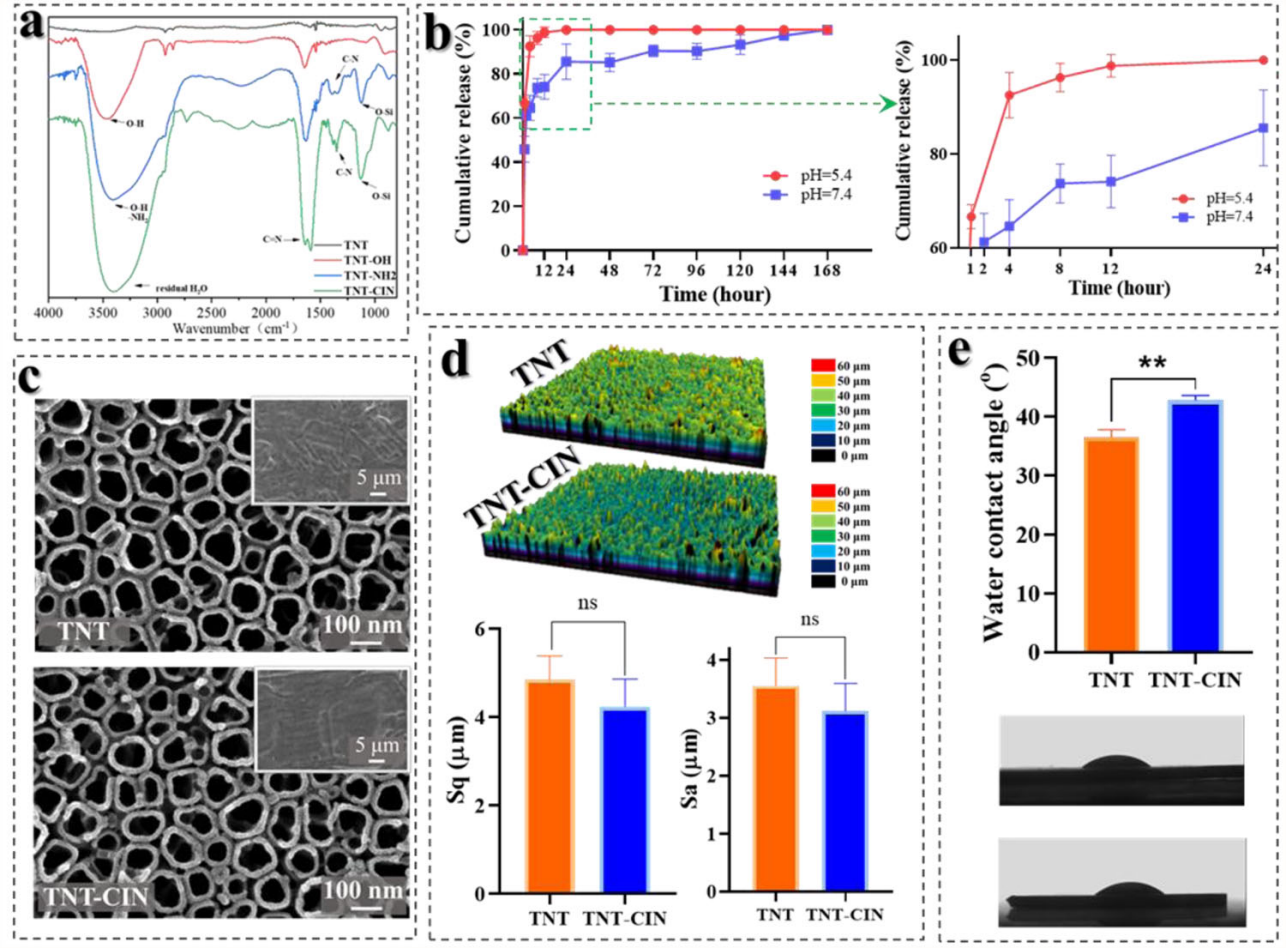
The characterization of TNT and TNT-CIN. **a** The FTIR result showed characteristic groups after each reaction step, indicating the successful synthesis of TNT-CIN. **b** Cumulative release curve of CIN showed that the acidic microenvironment enhanced the release rate of TNT-CIN in the first few hours. **c** The surface topography observed by SEM demonstrated that TNT-CIN retained the nanotube structure. **d** The roughness (Sa and Sq) did not significantly differ between TNT and TNT-CIN either. **e** The hydrophilicity of TNT-CIN was higher than that of TNT. **P < 0.01

The functional groups were detected to verify the successful bonding between TNT and CIN (Fig. 1a). The changes between steps in the synthesis process were analyzed. First, TNT-OH (TNT soaked in NaOH) was characterized by an absorption band in the region of 3200–3500 cm^−1^, which represented the O-H bond. Second, the bonds of -NH_2_, C-N, and O-Si were respectively detected with the absorption bands of 3400 cm^−1^, 1300 cm^−1^, and 1100 cm^−1^ after silylation reaction, indicating that TNT-NH_2_ was successfully constructed. The combination of TNT with CIN relied on the Schiff base reaction between the amino group of the treated TNT and the aldehyde group of CIN. Lastly, the absorption peak at 1600 cm^−1^ representing the Schiff base bond demonstrated that TNT-CIN had been successfully synthesized. Since hyperglycemia makes the microenvironment acidic, the experiment of CIN release was carried out in both acidic (pH = 5.4) and physiological conditions (pH = 7.4) to evaluate the pH response of TNT-CIN. The CIN concentration was detected at a specific time point, and then, cumulative release of CIN was calculated, which is shown in Fig. 1b. CIN was completely released within 12 h under acidic condition, whereas its complete release took over 168 h under physiological condition. In the first 1–24 h, CIN was released significantly faster in acidic condition than in physiological condition.

To ensure that TNT-CIN could retain the characteristics of nanotubes after the treatment, the surface topography, hydrophilicity, and roughness were analyzed. TNT and TNT-CIN were observed under a scanning electron microscope (Fig. 1c). At a lower magnification (2000×), TNT and TNT-CIN appeared relatively flat. At a higher magnification (100,000×), a nanotubular structure was evident on both their surfaces, indicating that binding CIN did not destroy the nanotube structure. Greater roughness was considered to be associated with stronger osseointegration. The roughness of TNT and TNT-CIN are shown in Fig. 1d. The average value of Sa was 3.55 ± 0.48 μm and 3.12 ± 0.48 μm (*n* = 3) for TNT and TNT-CIN groups, respectively. Even though the roughness of TNT-CIN was slightly lower than that of TNT, the difference was not significant (*P* = 0.33) as TNT-CIN still exhibited a neatly arranged and highly ordered nanotube structure. The Sq values shared the same tendency with Sa. Water contact angle measurement was used to evaluate the hydrophilicity of the solid materials, and the results are shown in Fig. 1e. The contact angle of TNT and TNT-CIN was 36.57 ± 1.27° and 42.8 ± 0.87° (*n* = 3), respectively. Although the water contact angle of TNT-CIN was marginally larger than that of TNT (*P* = 0.0021), both were demonstrated good hydrophilicity.

### TNT-CIN surface alleviated the inhibition on proliferation, spreading and osteogenic differentiation of BMSCs by HG and HG + LPS

**Fig. 2:**
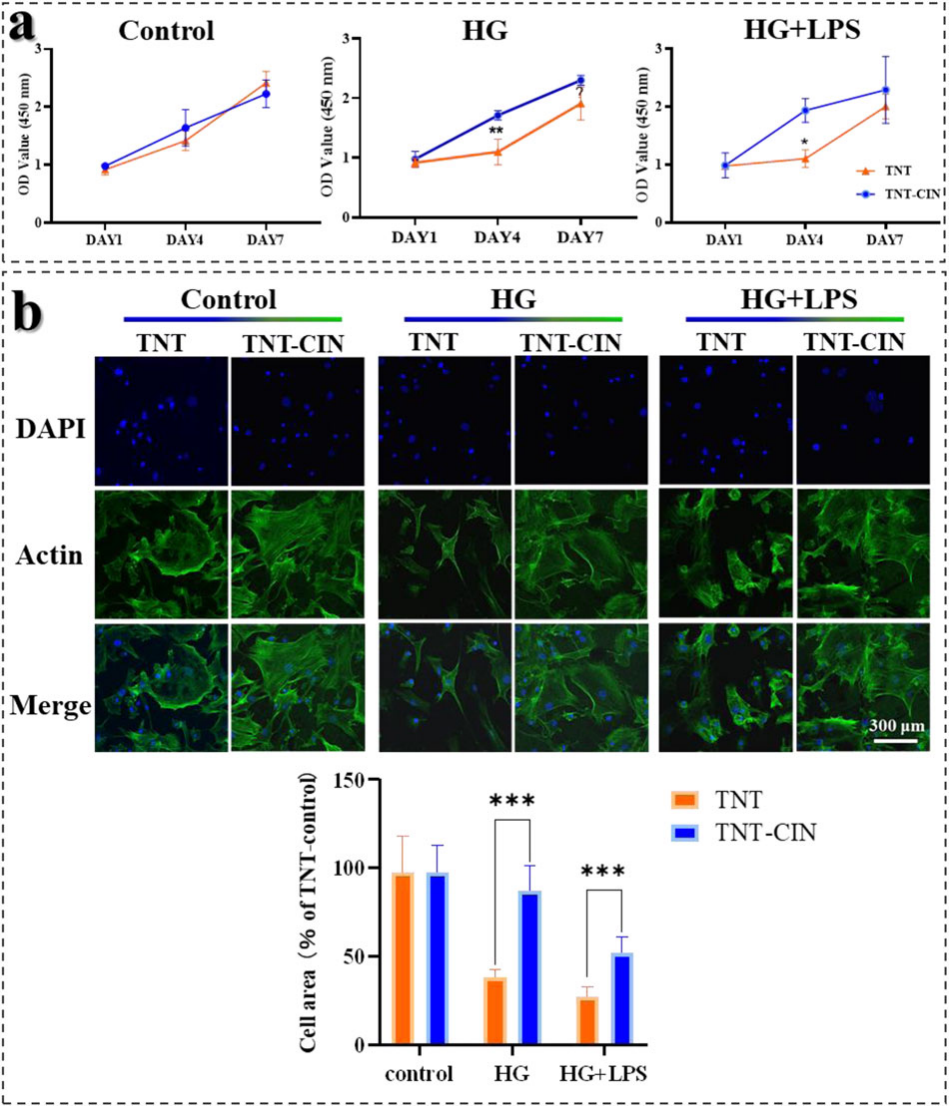
BMSCs on TNT-CIN showed preferable resistance to HG and HG + LPS as illustrated by better proliferation and spreading on TNT-CIN than on TNT. **a** The cell proliferation on TNT and TNT-CIN under different culture conditions after 1, 4, and 7 days. **b** The cytoskeleton staining under different culture conditions after 1 day as observed by confocal scanning laser microscopy and cell area analysis. *P < 0.05, **P < 0.01, ***P < 0.001. Scale bar = 300 μm

First, the cell proliferation and spreading properties were investigated to study the biological behavior under different conditions and on different substrates. As shown in Fig. 2a, BMSCs showed a time-dependent growth pattern on both surfaces under different conditions. On the fourth day, the viability of BMSCs was significantly higher on TNT-CIN than on TNT in HG (*P* = 0.0018) and HG + LPS conditions (*P* = 0.0119). Cell growth on TNT was evidently slowed down by HG + LPS exposure in the first four days. The result indicated that BMSCs showed better proliferation viability and resistance to HG and HG + LPS conditions on TNT-CIN than on TNT. The morphology of BMSCs on different titanium surfaces after 24-h culture is shown in Fig. 2b. Actin staining (green) revealed that the cells spread was more elongated and stretched in the control group than in HG and HG + LPS groups. However, cytoskeleton staining revealed that the cell area was larger in the TNT-CIN group than in the TNT group as shrunk BMSCs could be easily observed on TNT under HG and HG + LPS conditions (*P* < 0.001) in Fig. 2b.

**Fig. 3:**
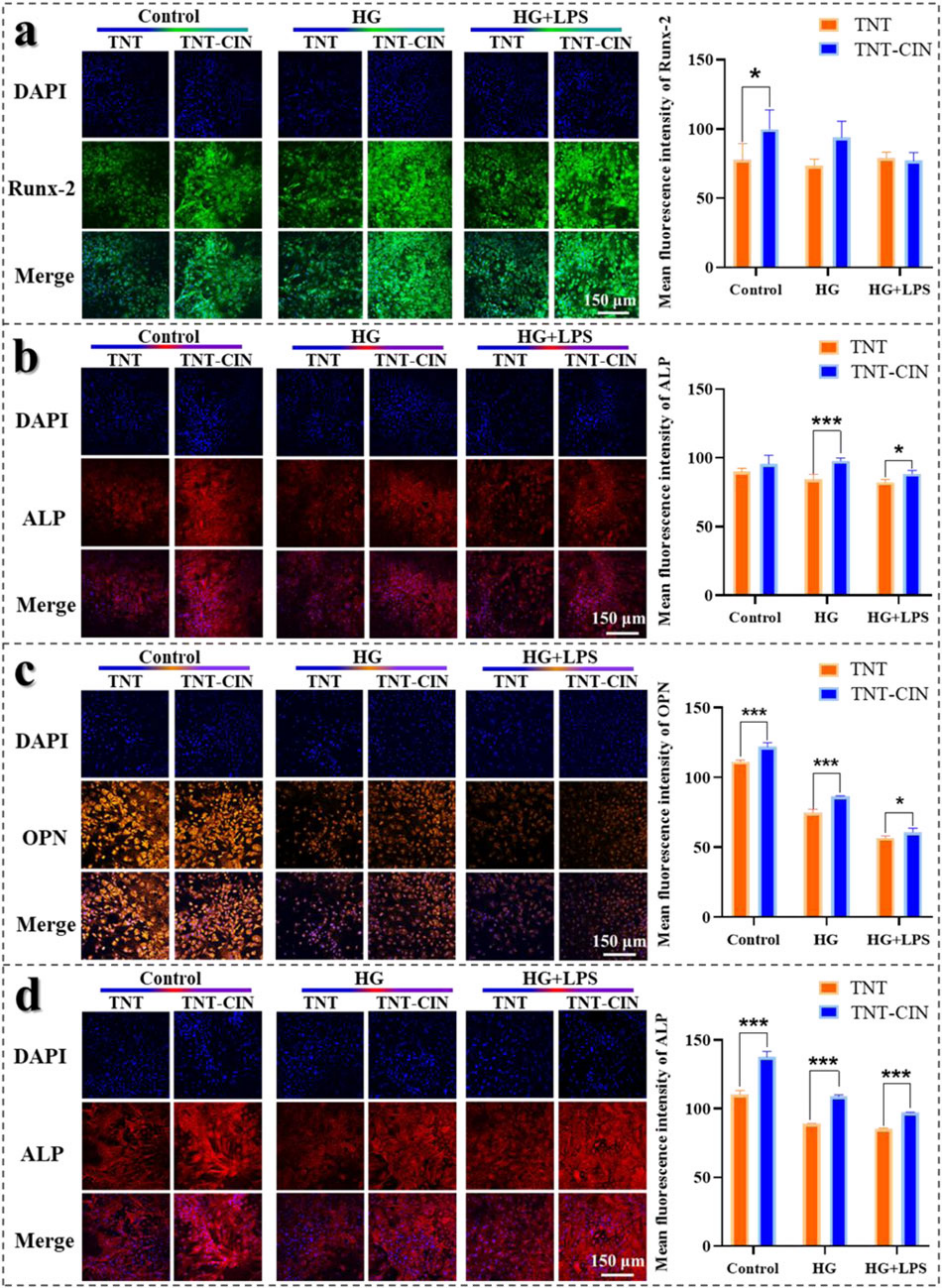
The expression of osteogenic proteins restrained by comprised condition appeared to be higher on TNT-CIN than on TNT. **a**, **b** The immunofluorescence staining of Runx-2 and ALP observed by confocal scanning laser microscopy after osteogenic induction for 7 days and its semi-quantitative analysis by Image J. **c**, **d** The immunofluorescence staining of OPN and ALP observed by confocal scanning laser microscopy after osteogenic induction for 14 days and its semi-quantitative analysis by Image J. *P < 0.05, **P < 0.01, ***P < 0.001. Scale bar = 150 μm

**Fig. 4:**
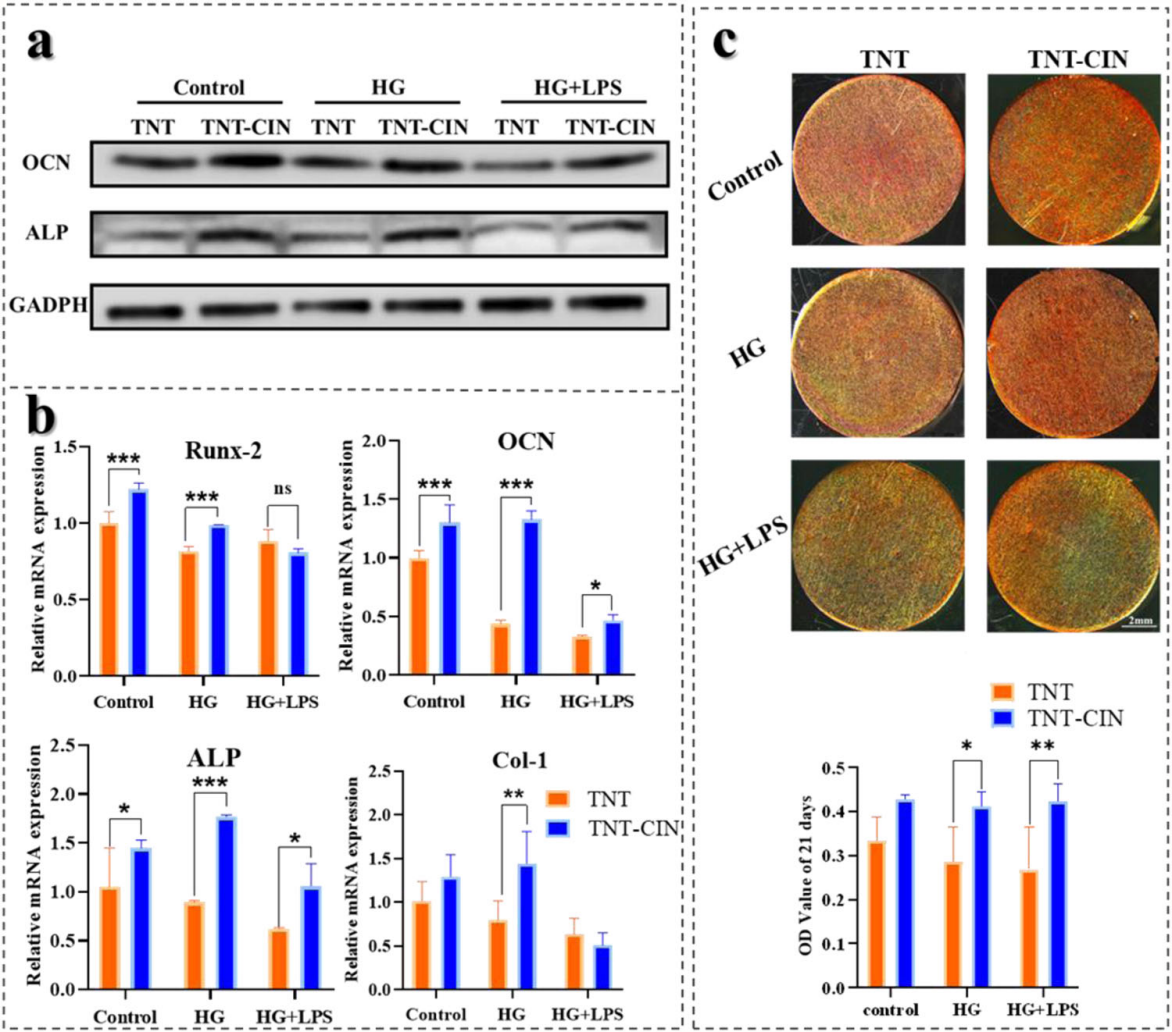
TNT-CIN showed excellent osteogenic ability illustrated by the increased expression of osteogenic proteins and mRNA and formation of calcified nodules. The osteogenic differentiation was restrained by HG, whereas HG + PG-LPS aggravated it. **a** The Western blot result after 14-day osteogenic induction on TNT and TNT-CIN. **b** The expression of mRNAs of osteogenic proteins on TNT and TNT-CIN after 14-day osteogenic induction. **c** The alizarin red staining on TNT and TNT-CIN under different conditions after osteogenic induction for 21 days and its semi-quantitative analysis by Image J. *P < 0.05, **P < 0.01, ***P < 0.001

Notably, HG + LPS conditions can inhibit osseointegration. To evaluate the osteogenic ability of TNT-CIN under diabetic conditions, the expression of proteins and their relative mRNAs was analyzed, and mineralization levels were detected. The expression of ALP, Runx-2, and OPN was observed using a confocal microscope after osteogenic induction for 7 and 14 days as shown in Fig. 3. After the 7-day osteogenic induction process, the expression of Runx-2 was significantly higher on TNT-CIN than on TNT in the control group (*P* = 0.0413) when ALP was significantly higher on TNT-CIN than on TNT in HG (*P* = 0.0005) and HG + LPS conditions (*P* = 0.0482). After the 14-day osteogenic induction process, the expression of OPN and ALP was higher on TNT-CIN than on TNT under all conditions (*P* < 0.0001, *P* < 0.0001, *P* = 0.0305 and *P* < 0.0001, *P* < 0.0001, *P* < 0.0001 respectively). This result showed an evident trend that HG and HG + LPS inhibited osteogenesis whereas TNT-CIN alleviated the inhibition of osteogenesis. In addition, the result of Western blot in Fig. 4a displayed similar tendency.

The relative mRNA expression of osteogenesis is shown in Fig. 4b. After osteogenic induction for 14 days, the expression of ALP, OCN, Runx-2 and Col-1 showed a decreasing trend in the following order: control > the HG group > the HG + LPS group, indicating that these compromised states inhibited the expression of osteogenesis-related genes. ALP and OCN are important markers to evaluate osteoblast differentiation, bone turnover, and bone formation. The expression of ALP and OCN was higher on TNT-CIN than on TNT in all conditions, whereas Runx-2 and Col-1 showed a similar trend without significant difference.

Mineralization levels were evaluated by alizarin red staining. As shown in Fig. 4c, TNT-CIN showed more calcific nodules under every condition at 21 days compared with TNT. Semi-quantitative analysis showed that the absorbance value of TNT-CIN was higher than that of TNT in HG group (*P* = 0.0224) and HG + LPS group (*P* = 0.0077), indicating that TNT-CIN had a positive effect on osteogenesis.

### TNT-CIN exerted anti-inflammation effect in HG and HG + LPS

Hyperglycemia can increase the inflammatory property of the microenvironment. The levels of ROS, an inflammation marker, were measured to evaluate the anti-inflammatory ability of TNT-CIN after stimulation with HG + LPS for 3 days. In this study, HG increased the oxidative stress, and LPS further exacerbated it (Fig. s1). Macrophages on TNT-CIN showed a lower ROS level than those on TNT, particularly in the HG group (*P* = 0.0373). In addition, the superoxide dismutase (SOD) activity in the HG + LPS group was evidently higher on TNT-CIN than on TNT (*P* = 0.0005). The increased SOD activity indicated that TNT-CIN exerted superior anti-inflammatory effect by scavenging ROS. The result confirmed that TNT-CIN was superior to TNT for attenuating the inflammation in the inflammatory microenvironment.

**Fig. 5:**
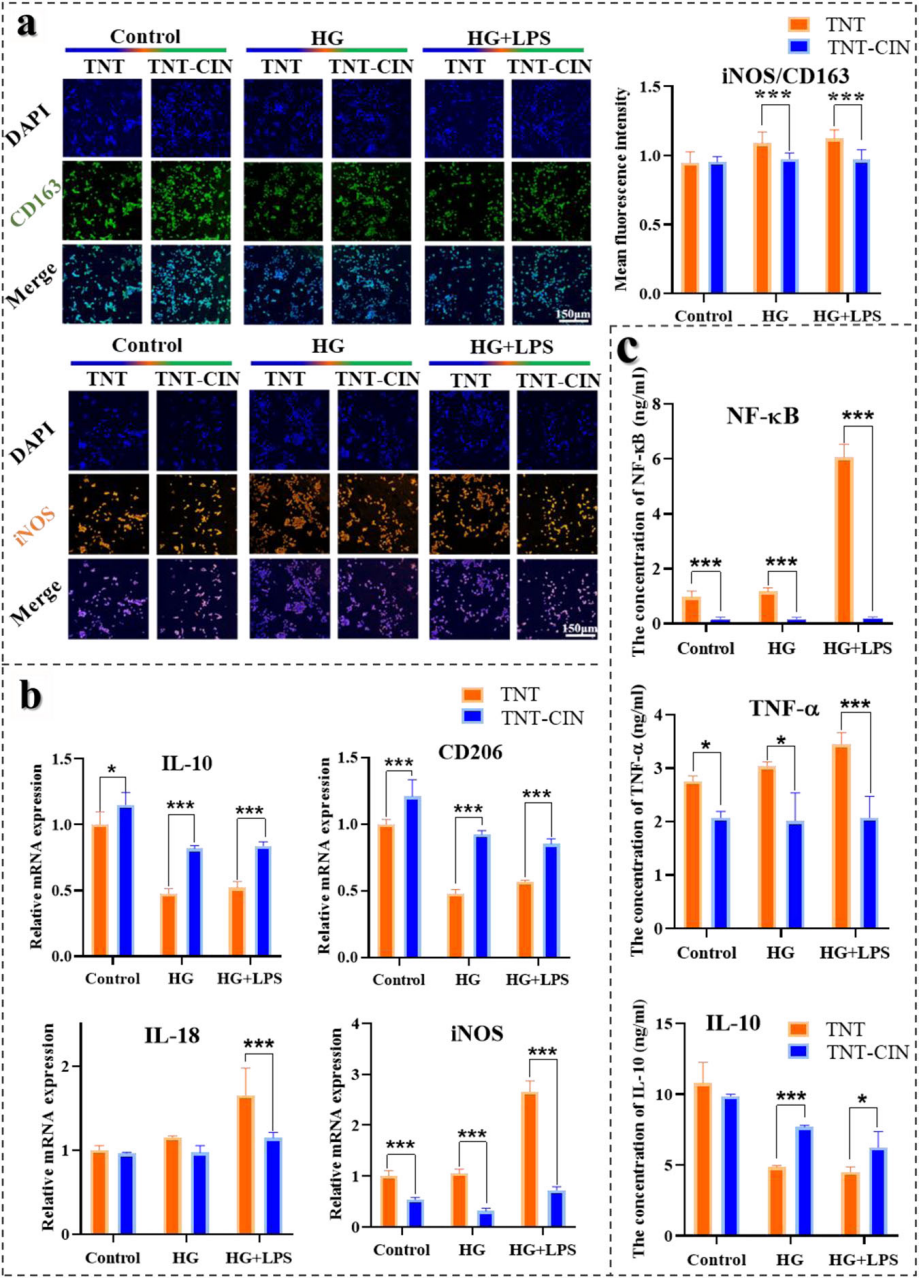
TNT-CIN induced the polarization of macrophages toward M2 and the secretion of fewer pro-inflammatory cytokines and more anti-inflammatory cytokines, indicating that TNT-CIN attenuated the inflammation caused by HG and PG-LPS. **a** The immunofluorescence staining of iNOS and CD163 observed by confocal scanning laser microscopy after 3 days and its semi-quantitative analysis by Image J. **b** The expression of relative mRNA under different culture conditions after 3 days. **c** The secretion of inflammatory cytokines under different culture conditions after 3 days. *P < 0.05, **P < 0.01, ***P < 0.001. Scale bar = 150 μm

Hyperglycemia can enhance inflammation by regulating the polarization of macrophages. It is believed that M1 type macrophages induce inflammation in and destruction of tissues, whereas M2 type macrophages promote reconstruction of tissues. To evaluate the polarization of macrophages, the expression of iNOS (M1 marker) and CD163 (M2 marker) was observed using a confocal microscope and analyzed semi-quantitatively (Fig. 5a). The results revealed that the iNOS:CD163 ratio was lower on TNT-CIN than on TNT in HG (*P* = 0.001) and HG + LPS groups (*P* < 0.0001), indicating more macrophages of M2 type adhered to TNT-CIN, whereas more macrophages of M1 type adhered to TNT. Similarly, the results of the relative mRNA expression of IL-10, CD206, IL-18, and iNOS showed the same tendency (Fig. 5b). Compared with TNT, TNT-CIN showed lower expression of pro-inflammatory mRNA expression, such as IL-18, and iNOS, and increased anti-inflammatory cytokines, such as CD206 and IL-10. These results indicated TNT-CIN transformed more macrophages into an anti-inflammatory and repairing state compared with TNT.

Since macrophages regulate immune response by synthesizing and secreting cytokines, the inflammatory and anti-inflammatory cytokines were measured on the third day to evaluate the reaction of macrophages in an inflammatory environment on different Ti surfaces (Fig. 5c). TNF-α and NF-κβ, which are largely involved in systemic inflammation and induce more pro-inflammatory cytokines, were found to be secreted less on TNT-CIN than on TNT, whereas IL-10, which is an anti-inflammatory cytokine, was founded to be secreted more on TNT-CIN than on TNT in the HG and HG + LPS group. It was obvious that HG and HG + LPS groups showed greater secretion of pro-inflammatory cytokines on TNT but negligible secretion of pro-inflammatory cytokines on TNT-CIN, indicating the TNT-CIN could resist inflammation better than TNT.

### TNT-CIN had superior ability to resist *S. mutans* and *P. gingivalis*

**Fig. 6:**
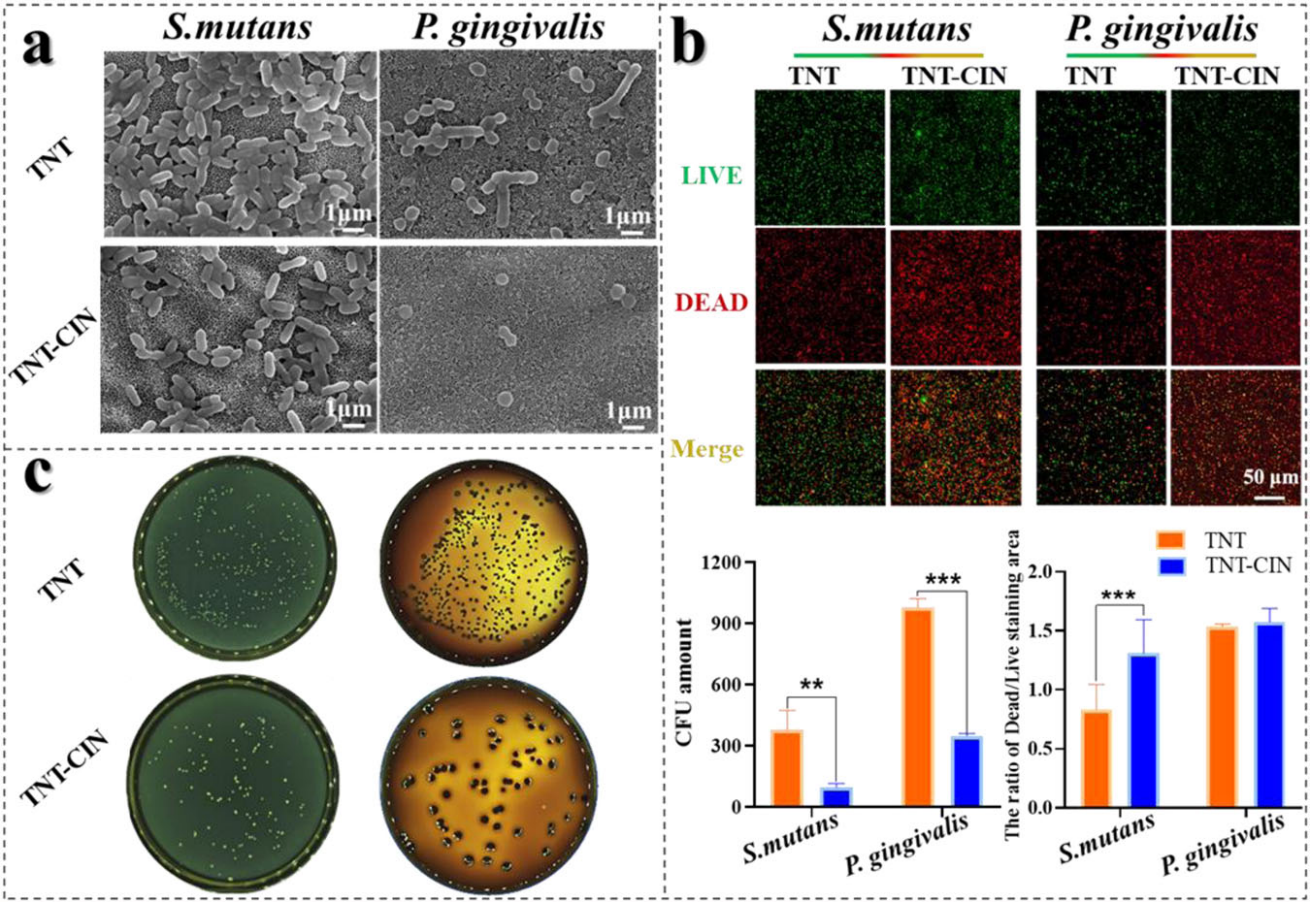
TNT-CIN strongly resisted *S. mutans* and *P. gingivalis*. **a** Cells subjected to LIVE/DEAD staining observed by confocal microscope after culturing on different surfaces for 1 day and its semi-quantitative analysis. **b** *S. mutans* and *P. gingivalis* adhered on TNT and TNT-CIN after 1 day, as observed by SEM. **c** The viable *S. mutans* and *P. gingivalis* on different surfaces after 1 day (diluted 1000 folds) and its quantitative analysis. **P < 0.01, ***P < 0.001. Scale bar = 50 μm

Peri-implantitis continues to be one of the main causes of implant failure. Once a mature biofilm has developed on any implant surface, bacterial eradication becomes highly challenging even with the use of antibiotic therapy and repeated surgical irrigation and debridement. The prevention of biofilm formation is the best way to avoid the spread of pathogens. *S. mutans* is the first to attach to the surface of the tooth in the process of biofilm formation. *P. gingivalis* is the predominantly cause of peri-implant inflammation. Therefore, the ability to resist the growth of these species was evaluated in this study. As observed by SEM (Fig. 6a), evidently fewer *S. mutans* could adhere on TNT-CIN than on TNT, on which stacked *S. mutans* was found. Meanwhile, *P. gingivalis* appeared to have shrunk on TNT-CIN, whereas it retained its typical, rod-like shape on TNT. LIVE/DEAD staining showed dead bacteria in red when alive bacteria in green. The fluorescence images and semi-quantitative analysis showed more dead and fewer alive *S. mutans* on TNT-CIN than on TNT (Fig. 6b; *P* = 0.0005). The results of plate coating demonstrated that the number of viable *S. mutans* and *P. gingivalis* decreased to a greater extent on TNT-CIN than on TNT (Fig. 6c; *P* = 0.0066, *P* < 0.0001 respectively). These evidences indicated that TNT-CIN had better resistance to bacteria adhesion and biofilm formation than TNT.

## Discussion

Currently, dental implant treatment has been widely accepted as an effective method to resolve dentition defects/loss. However, the wound in patients with diabetes takes longer to heal and is susceptible to infection. This results in defects in osseointegration and a higher implant failure rate due to premature loss of the implant [32]. Therefore, diabetes has been considered a challenging obstacle and remains a relative contraindication for implant therapy.

Considering these challenges, there has been increasing scientific focus on surface modifications that can improve implant osseointegration in patients with diabetes. A few bioactive coatings on a porous Ti substrate have been constructed and demonstrated to improve osteogenesis in animal models of diabetes, such as tantalum [33] or PLGA coating [34]. Nowadays, nano-scale topography has attracted much attention as its size being similar to that of the cell receptor can easily influence the cell fate by targeting receptor-driven pathways [35]. Therefore, nano-scale hydroxyapatite has been applied in combination with chitosan or silicon in patients with diabetes, and this approach has proven to promote bone formation [36]. However, the above coating did not have a regular or controllable nanotopography. TiO_2_ nanotube array (TNT), which has a regular and highly ordered topography, is easy to adjust the tube diameter and length by altering the voltage, time, and electrolytes, among other aspects. In our previous study, TNT was demonstrated to improve osteogenesis in normal healthy individuals [10] and was found to alleviate diabetes-induced osteogenic inhibition [11]. It was further found that TNT induced osteoimmunal regeneration under pathological conditions of oxidative stress by inducing FoxO1 signaling pathways [31]. Meanwhile, owing to their tubular structure, TiO_2_ nanotubes had high surface-to-volume ratio, which has been considered as a favorable trait for a drug carrier. However, most applications of nanotube for drug delivery either impair the nanotopography of TNT itself or require a complex synthesis process [37]. Silanization, on the other hand, has been widely used in surface modification to provide an amino group, which helps binding molecules to the substrates. The method is easily accomplished and reportedly to preserve the nanotube structure [12].

As for the grafted chemicals, the mild plant-derived extracts have gradually attracted the attention of scientists as they are more cost effective and safer than most bioactive factors [25]. CIN is the main active ingredient of cinnamon oil, which is a type of plant extract. It is a yellow viscous liquid with strong aroma of cinnamon oil and a mild spicy smell. CIN comprises an acrolein and a phenyl group, and its simple formula is C_6_H_5_CHCHCHO. CIN first drew public attention for its hypoglycemic effect. It was shown to lower glucolipid levels in diabetic animals via multiple signaling pathways, such as PPARs, AMPK, PI3K/IRS-1, and RBP4-GLUT4 [23]. Emerging evidences from further studies proved that CIN had multiple effects. CIN increased osteogenesis and induced bone formation by promoting the differentiation of osteoblasts and prohibiting the activity of osteoclasts in ovariectomized rats, suggesting that it could be a candidate compound to improve osteogenesis and may play an important role in the treatment of osteoporosis clinically [24, 26]. CIN was also reported to have a strong effect on decreasing oxidative stress through many signaling pathways, such as Nrf2 signaling, TGF-β1, IL-13-mediation, and the TLR4–NOX4/IRAK4 pathway [22, 38, 39]. In addition, CIN damaged bacterial permeability and membrane integrity because of its hydrophobic nature and its interaction with the lipid bilayer of cytoplasmic membranes [20, 29], which caused bacterial lysis and death via the inhibition of electron transport chain, protein translocation, and cellular component synthesis [40]. Notably, CIN also inhibited the biofilm formation of *P. gingivalis* dose-dependently [20]. The advantage of CIN is that it supports cell survival over a wide safety range; however, at low concentrations, it leads to death of bacteria. Briefly, CIN has anti-inflammatory, antibiotic, and osteogenic effects.

In this study, we combined TNT with CIN to improve the properties of the implant for patients with diabetes. As shown in Fig. 2c, TNT-CIN preserved the nanotube structure of TNT, indicating that TNT-CIN could exert the effects induced by nanotopography. Even though there was a marginal difference in water contact angle between TNT and TNT-CIN (Fig. 2d), both had hydrophilic property, which is defined by a water contact angle of less than 90°; furthermore, this slightly lowered water contact angle indicated that CIN was loaded onto TNT as CIN contained a hydrophobic benzene ring. However, the hydrophilicity was not decreased greatly by the hydrophobic benzene ring, which could be due to the hydrophilicity of the residual amino group on TNT-CIN. The combination of TNT with CIN relied on the Schiff base bond between the amino group of the treated TNT and the aldehyde group of CIN. The hydrolysis of the Schiff base bond could be accelerated in an acidic environment. This mechanism has been widely used to trigger faster release of anti-cancer drugs in acidic tumor environment [41]. Reports have shown that the pH values of microenvironment in diabetes and inflammation could reduce to 6.6 and 5.4, respectively, both indicating an acidic microenvironment [30, 42].

As shown in a cumulative release curve, in the first 1–24 h, CIN was released faster in an in vitro environment with a pH of 5.4 than in one with a pH of 7.4 because the amide bond was easily hydrolyzed in the acidic solution. This evidence not only demonstrated the existence of a chemical bond between CIN and TNT but also confirmed that TNT-CIN exhibited a pH-responsive pattern, suggesting that the acidic environment induced by diabetes may accelerate the release of CIN. The cumulative amount of CIN released in 7 days from TNT-CIN was in the safe range for osteogenesis and reached a concentration at which CIN can exert anti-inflammatory (1 μM) [39] and antibiotic (2.5 μM) effects [20]. The research [43] revealed three distinct phases when cells adopt a committed expression phenotype: initiation of differentiation (0–3 h, phase I), lineage acquisition (6–24 h, phase II), and early lineage progression (48–96 h, phase III). The early release of CIN help BMSCs establish osteogenic phenotype. In the process of wound healing, monocytes gather and differentiate into macrophages at the 24^th^ hour, and then polarize toward M1 or M2 type. M1 macrophages are dominant in the early stage resulting in inflammation, while M2 macrophages take 5–7 days to reach 80–85% of the number of macrophages leading to proliferation. Diabetes will prolong the time of phenotypic transformation [44]. Therefore, the early release of CIN can affect the polarization direction of macrophages and play an important role in wound healing. In terms of anti-infection, a retrospective study [45] observed the early period from the implantation to the connection of prostheses and found that the risk of implantation failure multipled by 1.1 once the postoperative infection ocurred every week earlier. It was believed that the early application of antibiotics was conducive to the delayed occurrence of implant infection. Therefore, the early release of CIN in this experiment was conducive to reducing the possibility of failure of implant. In addition, the in vivo bioactivity of cinnamaldehyde came not only from itself but also from its metabolites, which alleviated the concern of CIN metabolizing into other ineffective products [23].

The most common method to simulate diabetes in vitro was adjusting glucose concentration. Previously, 22 mM glucose was demonstrated to inhibit proliferation, adhesion and osteogenesis strongly, [11]. In this experiment, HG group (22 mM glucose) similarly showed inhibition on proliferation, adhesion, osteogenesis of BMSCs and increased level of inflammation of macrophages. Then in order to mimic the infection on the basis of diabetes, 1 μg/mL *P.gingivalis*-LPS was used. *P.gingivalis*-LPS was widely used in the establishment of in vitro models of peri-implant infection, when 1 μg/mL was demonstrated to alleviate osteogenesis but do not inhibit proliferation [46]. In Fig. 3a, b, the inhibition caused by HG and HG + LPS was not obvious, which might be explained by the reason that the formation of chronic inflammatory environment needed to take some time. The other research [47] found that the reparative osteogenesis of chronic hyperglycemia at the 14^th^ day had signs of inflammation which characterized by inflammatory cell infiltration occupied 8.37% of the entire osteoreparation zone and granulation tissue area 9.99%, while at the 30^th^ day the component of reparative osteogenesis turned to bone tissue and cartilage tissue. It meant that the inflammatory effect of high glucose could be more obvious at the 14^th^ day compared to the 30^th^ day. Therefore, whether the model successfully established could be evaluated at 14-day osteogenic induction. In this experiment, the osteogenesis after 14-day osteogenic induction was obviously inhibited by HG and HG + LPS (Fig. 3c, d), which was consistent with above research. In addition, macrophages are the most important cells to react to inflammation, of which the ROS level, the expression of M1 marker, and the secretion of proinflammatory cytokines also increased in HG and HG + LPS conditions. Briefly, the model of diabetes and diabetes with infection were established successfully.

Kania et al. [48] found that cinnamon increased the levels of bone turnover markers in ovariectomized rats and induced the granular shape of the tibia. Lee et al. proved that cinnamon bark extract increases the viability of MC3T3-E1 cells and ALP activity and that it inhibits the production of IL-6 and nitric oxide in the presence of TNF-α, indicating it can stimulate bone formation in vitro and prevent osteoporosis and inflammatory bone diseases [49]. CIN was further investigated to identify if it inhibits the formation of osteoclasts and promotes the formation of osteoblasts in the treatment of glucocorticoid-induced osteoporosis [25]. Consequently, it was found that CIN may be used as a drug for the treatment of osteoporosis. In this study, the proliferation and osteogenic differentiation of BMSCs on TNT-CIN showed similar osteogenic ability to the above study, particularly with regard to the evidently better performance than TNT in the simulated diabetes conditions (Fig. 3c, d). Bone formation mainly goes through four stages: osteoblast proliferation, maturation of extracellular matrix, mineralization of extracellular matrix and apoptosis of osteoblast [50]. Runx-2 is critical for osteogenesis in early stage, which triggers the formation of bone matrix protein but prevents osteoblasts from further maturation [51]. The expression of Col-1 is gradually declined when mineralization began [50]. In Fig. 4b, the expression of Runx-2 and Col-1 on TNT-CIN was slightly lower than TNT in HG + LPS, which could be explained by that CIN exerted strong anti-inflammatory effect under aggravated inflammation and accelerate the process of osteoblast maturation under the condition of HG + LPS. However, the mechanism of osteogenic regulation involving Runx-2 is very complicated. OCN is the maturation marker of osteoblast. The expression of OCN was higher on TNT-CIN under HG + LPS also demonstrated that TNT-CIN under HG + LPS helped to accelerate bone formation. It was also possible that TNT-CIN had potential mechanisms to promote the expression of ALP and OCN. The result of alizarin red staining also demonstrated the mineralization on TNT-CIN was better.

Besides, the immune microenvironment for osteogenesis is critical. Diabetes delays macrophages from M1 type to M2 type and prolongs the inflammatory phase, resulting in a chronic, unresolving inflammation of the injured tissue, which damages osteogenic ability. In this study higher M2 marker expression, higher antioxidant capacity, and lower ROS level on TNT-CIN suggested that TNT-CIN could reduce inflammatory reaction and accelerate the transformation of macrophages from M1 type to M2 type. TNF-α and NF-κβ are considered as key factor for activation of osteoclastic differentiation [52]. The secretion of TNF-α and NF-κβ was lower on TNT-CIN, which was consistent with the report that CIN inhibited the expression of TNF-α, NF-κβ, and IL-1, among other cytokines [53]. The research [54] reported that IL-10 secreted by macrophages could help osteogenesis in a dependent manner. In this study, the cytokines IL-10 was higher on TNT-CIN. These results (Fig. 5c) indicated osteogenesis was improved while osteoclastogenesis was inhibited by the secretion of macrophages on TNT-CIN. The report used homology modeling technique and docking simulation studies to indicate that CIN interacted with TLR4 and NOX4 through hydrogen bond interactions [39]. LPS recognition was predominantly mediated by TLR4, leading to inflammation by activating downstream cascades. Therefore, TNT-CIN was hypothesized to compete with LPS in binding with TLR-4, thus resulting in the decreased level of inflammation.

The accumulation of bacteria and formation of plaque before integration between the implant and the surrounding soft tissue is the main cause of early implant failure [55]. Considering that hyperglycemia increases the possibility of opportunistic infections and the difficulty of killing bacteria after implant surgery, strategies preventing biofilm formation on the implant become extremely important. Since *S. mutans* is the first bacteria to adhere to surface, and *P. gingivalis* is the main pathogen behind peri-implant inflammation, the resistance of TNT-CIN to *S. mutans* and *P. gingivalis* was evaluated in this study. Compared with TNT, TNT-CIN led to morphological shrinkage of *P. gingivalis*, which is a manifestation of bacterial inactivation. The antibacterial rate of TNT-CIN was 74.7% against *S. mutans* and 64.3% against *P. gingivalis* as calculated by the number of bacterial colonies when TNT was regarded as the control group. The antibacterial rate of both ranged between 50% and 90%, indicating that TNT-CIN had antibacterial properties. A previous study reported the leakage of RNA and DNA and demonstrated that CIN impaired the membrane integrity of *P. gingivalis* by enhancing membrane permeability [20]. CIN can diffuse and destabilize the polysaccharide matrix of the biofilm. Jia et al. demonstrated decreased expression of the biofilm-related gene sarA in methicillin-resistant *Staphylococcus aureus* when exposed to CIN [56]. The review also illustrated that the antibacterial ability of CIN may be attributed to the alteration of lipid profile, its anti-quorum sensing effects, and the inhibition of ATPases, cell division, membrane porins, motility, biofilm formation [57]. These mechanisms explain the antibacterial effect of TNT-CIN in this study.

TNT-CIN exhibited excellent osteogenic ability in the simulated diabetes condition. This could be attributed to the anti-inflammatory and antibacterial effects of TNT-CIN which improved the impaired microenvironment caused by diabetes. In summary, TNT-CIN is a multifunctional coating which was found to exert osteogenic, anti-inflammatory, and anti-bacterial properties in the simulated diabetes condition.

## Conclusion

In this study, a pH-responsive cinnamaldehyde–TiO_2_ nanotube coating was successfully constructed. TNT-CIN was demonstrated to have similar characteristics as TNT and was found to release CIN in a pH-responsive manner. Compared with TNT, TNT-CIN improved osteogenesis and promoted anti-inflammation in the diabetes-stimulated condition and showed better antibacterial ability as well. These results indicate that TNT-CIN can be considered to have application prospects in patients with diabetes requiring dental implants.

## Supplementary Information

Supplementary Information
