# Supplementary material for: pH-responsive cinnamaldehyde-TiO2 nanotube coating: fabrication and functions in a simulated diabetes condition
Source: J Mater Sci Mater Med. 2022 Sep 5;33(9):63. doi: 10.1007/s10856-022-06683-2 (PMC9444834; doi:10.1007/s10856-022-06683-2)
Supplement: Supplementary file 1 — Supplementary Information [file 10856_2022_6683_MOESM1_ESM.docx]

Supporting Information for:

**pH-responsive cinnamaldehyde TiO_2_ nanotube coating: fabrication and multiple functions in a simulated diabetes condition**

Yichen Lee ^a,b,1^, Jingyan Huang ^a,b,1^, Zhaoxia Bing ^a,b^, Kaiting Yuan ^a,b^, Jinghong Yang ^a,b^, Min Cai ^a,b^, Shiqi Zhou ^a,b^, Bo Yang ^a,b^, Wei Teng ^a,b^, Weichang Li ^a,b,^*, Yan Wang ^a,b,^**

**Table. s1** Primer sequences

| Gene | Forward primer sequence（5’-3’） | Reverse primer sequence (5’-3’) | |
| --- | --- | --- | --- |
| GAPDH | TGACCACAGTCCATGCCATC | | GACGGACACATTGGGGGTAG |
| Runx-2 | GGGAACCAAGAAGGCACAGA | | ACTTGGTGCAGAGTTCAGGG |
| OCN | CCCTGAGTCTGACAAAGCCTTCA | | AGATGCGTTTGTAGGCGGTC |
| ALP | CCAACTCTTTTGTGCCAGAGA | | GGCTACATTGGTGTTGAGCTTTT |
| Col-1 | GGCTACATTGGTGTTGAGCTTTT | | TCCAAACCACTGAAGCCTCG |
| iNOS | CACCAAGCTGAACTTGAGCG | | CGTGGCTTTGGGCTCCTC |
| IL-18 | TGGCCGACTTCACTGTACAAC | | TGGGGTTCACTGGCACTTTG |
| CD206 | AGACGAAATCCCTGCTACTG | | CACCCATTCGAAGGCATTC |
| IL-10 | TAAGGCTGGCCACACTTGAG | | GTTTTCAGGGATGAAGCGGC |


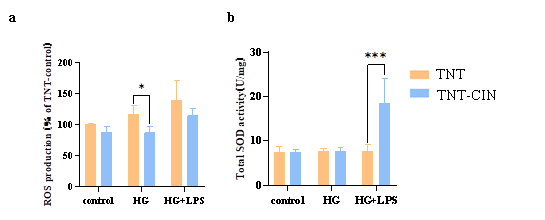


**Fig. s1** TNT-CIN decreased the ROS level caused by high glucose and high glucose with LPS, which may attribute to the increased SOD activity, especially under the condition of 22 mM with LPS (**a**)The ROS production of TNT and TNT-CIN under different conditions. (**b**) The total SOD activity of TNT and TNT-CIN under different conditions. ** P*< 0.05, ****P*< 0.001
